# Regions of KCNQ K^+^ channels controlling functional expression

**DOI:** 10.3389/fphys.2012.00397

**Published:** 2012-10-16

**Authors:** Frank S. Choveau, Mark S. Shapiro

**Affiliations:** Department of Physiology, University of Health Science Center at San AntonioSan Antonio, TX, USA

**Keywords:** potassium channels, KCNQ, Kv7, gating, structure/function

## Abstract

KCNQ1–5 α-subunits assemble to form K^+^ channels that play critical roles in the function of numerous tissues. The channels are tetramers of subunits containing six transmembrane domains. Each subunit consists of a pore region (S5-pore-S6) and a voltage-sensor domain (S1-S4). Despite similar structures, KCNQ2 and KCNQ3 homomers yield small current amplitudes compared to other KCNQ homomers and KCNQ2/3 heteromers. Two major mechanisms have been suggested as governing functional expression. The first involves control of channel trafficking to the plasma membrane by the distal part of the C-terminus, containing two coiled–coiled domains, required for channel trafficking and assembly. The proximal half of the C-terminus is the crucial region for channel modulation by signaling molecules such as calmodulin (CaM), which may mediate C- and N-terminal interactions. The N-terminus of KCNQ channels has also been postulated as critical for channel surface expression. The second mechanism suggests networks of interactions between the pore helix and the selectivity filter (SF), and between the pore helix and the S6 domain that govern KCNQ current amplitudes. Here, we summarize the role of these different regions in expression of functional KCNQ channels.

## Introduction

KCNQ1–5 genes encode five different voltage-gated K^+^ channels, consisting of tetramers, with each subunit containing six transmembrane domains (S1–S6), divided between a voltage-sensor domain (S1–S4), a pore domain (S5-pore-S6) and a long cytoplasmic carboxy terminus. These channels play critical roles in the heart, ear, nerves, and epithelial tissue (Robbins, 2001) and display sharply divergent current amplitudes. Thus, KCNQ2 and KCNQ3 yield small currents compared to KCNQ1 and KCNQ4 (Kubisch et al., 1999; Gamper et al., 2003, 2005; Schenzer et al., 2005; Schwake et al., 2006), although their unitary conductance is greater than those of KCNQ1 or KCNQ4 (Pusch, 1998; Selyanko et al., 2001; Li et al., 2004). Moreover, co-expression of KCNQ2 and KCNQ3 to form KCNQ2/3 heteromers results in a >10-fold increase in current amplitudes compared to KCNQ2 or KCNQ3 homomers (Kubisch et al., 1999; Gamper et al., 2003). Three different regions have been suggested as responsible for such divergent current amplitudes: the N-terminus, the pore region, and the C-terminus. The N-terminus has been shown to play a role in channel trafficking (Dahimene et al., 2006) and open probability (Etxeberria et al., 2004). The pore domain contains two networks of interactions governing the channel conductive pathway. The first involves hydrogen bonds between the pore helix and the selectivity filter (SF) (Zaika et al., 2008; Choveau et al., 2012a). The second implicates van der Waals interactions between the S6 domain and the pore helix (Seebohm et al., 2005; Choveau et al., 2012b). Finally, four helices within the C-terminus are required for the channel to function properly. Helices A and B are critical for channel modulation by the ubiquitous Ca^2+^ sensor, calmodulin (CaM) (Wen and Levitan, 2002; Yus-Najera et al., 2002; Gamper and Shapiro, 2003; Gamper et al., 2005; Etxeberria et al., 2008; Haitin and Attali, 2008; Alaimo et al., 2009; Etzioni et al., 2011). The distal helices C and D, required for channel assembly and tetramerization, seem also involved in channel surface expression (Schwake et al., 2003; Etxeberria et al., 2004; Howard et al., 2007) as is the N-terminus. Here, we summarize the major mechanisms suggested to regulate the surface expression and current amplitudes of KCNQ channels.

## The N-terminus plays a role in channel trafficking and open probability

Mutagenesis studies have suggested the N-terminus to play a role in the localization of KCNQ channels at the membrane. Two motifs within the N-terminus of KCNQ1 have been identified as being critical. The first motif, leucine-glutamate-leucine, is located at positions 38–40. Substitution of leucines to alanines in this motif induced retention of KCNQ1 channels in endosomal compartments of polarised epithelial MDCK cells (Jespersen et al., 2004). The second involves a tyrosine at position 51. Replacement of this tyrosine with an alanine led to a relocalisation of channels from the basolateral to the apical membranes. Moreover, insertion of 17 residues, including this tyrosine, into a neurotrophin receptor, led to a relocalisation of this receptor from the apical to basolateral membranes, suggesting that the N-terminus influences channel trafficking. Consistent with this, serial deletions in the N-terminus of KCNQ1 revealed the first 106 residues to not affect KCNQ1 surface expression, whereas deletion of the first 114 residues reduced it (Dahimene et al., 2006), suggesting a role of residues 106–114 in channel surface expression. Interestingly, three mutations (Y111C, L114P, and P117L) in this region, are related to the long-QT syndrome of cardiac arrthymias (Splawski et al., 2000; Schwartz et al., 2001; Jongbloed et al., 2002). Membrane expression assays were performed to investigate the effect of those mutations on channel trafficking. None of the mutants were detected at the plasma membrane by immunofluorescence in CHO cells or in cardiomyocytes (Dahimene et al., 2006). Moreover, the Y111C mutation led to a retention of channels in the endoplasmic reticulum (ER), suggesting that those residues are part of an ER export signal. However, insertion of a fragment (106–114) containing those residues in the Y111C mutant did not restore normal channel trafficking, arguing against a role of those residues as an export signal. A sequence alignment of KCNQ1–5 shows a conserved motif in the N-terminus **Y**XX**L**ER**P**XGW, including Y111, L114, and P117 (in bold), suggesting a similar role of those residues in other KCNQ subunits. Indeed, replacement of the analogous leucine (L85) in KCNQ2 with a proline completely abolished surface expression (Dahimene et al., 2006), reinforcing the role of the N-terminus in channel surface expression, a role that deserves further study.

However, another study reported the N-terminus to mostly affect the open probability of KCNQ2/3 channels rather than channel trafficking to the membrane (Etxeberria et al., 2004). Replacement of the N-terminus of KCNQ2 by that of KCNQ3 led to a 12-fold increase of current amplitudes when co-expressed with KCNQ3, whereas surface expression was modestly decreased. Consequently, this increase of current amplitude cannot be explained by a modification of surface expression. Moreover, substitution of the N-terminus of KCNQ3 by that of KCNQ2 decreased current amplitudes when the chimeras were expressed with KCNQ2 (Etxeberria et al., 2004), indicating the N-terminus may control open probability. The lower impact of the N-terminus on KCNQ2/3 surface expression compared to KCNQ1 could be due to the weak conservation of this region between those channels. In conclusion, the N-terminus may control KCNQ channel open probability and/or surface expression, depending on the specific channel. However, the C-terminus, which is required for assembly, has also been suggested critical for surface expression, as described below (Schmitt et al., 2000; Schwake et al., 2003, 2006; Etxeberria et al., 2004).

## Role of the C-terminus in controlling channel surface expression

Many studies have investigated the role of the C-terminus on channel function. According to those studies, the divergent current amplitude of KCNQ channels results from differential surface expression of channels controlled by the distal part of the C-terminus. Thus, introduction of the C-terminus of KCNQ1 by that of KCNQ3 enabled the chimeric subunit to form heteromers with KCNQ2, resulting in an increase of current caused by a greater number of chimeric channels at the plasma membrane, compared to KCNQ1 (Schwake et al., 2003). The larger current amplitudes of KCNQ2/3 heteromers, compared to KCNQ2 or KCNQ3, would then be due to increased surface expression of channel proteins (Schwake et al., 2000). Using the oocyte expression system, the relevant part of the C-terminus of KCNQ1-3 channels was suggested to encompass both the C and D helices, termed the “subunit-interaction domain” (Schwake et al., 2006), and a similar result was found in hippocampal neurons for KCNQ2 and KCNQ3 (Chung et al., 2006). In particular, the D-helix has been suggested as specifically involved in KCNQ channel tetramerization (Howard et al., 2007). Thus, substitution of the D-helix of KCNQ2 by that of KCNQ1 enhanced KCNQ2 currents and surface expression (Schwake et al., 2006), suggesting a correlation between channel surface expression and current amplitude. The crystal structure of the D-helix of KCNQ4 has suggested channel oligomerization to be promoted by formation of salt bridges between residues of opposite charge in the D-helix, which are divergent in KCNQ3 (Howard et al., 2007). KCNQ3 channels possess four phenylalanines (one from each monomer) at position 622 instead of the valines in KCNQ4–5 or leucines in KCNQ1–2, which prevent formation of a stable tetramer when assayed *in vitro*. Indeed, C-termini bearing the F622L mutation were found to be predominantly tetrameric. Biochemical analysis of the D631/SG633E double mutation, which restores salt bridges in the D-helix, also resulted in a stabilization of KCNQ3 C-termini into a tetrameric form; however, augmentation of current amplitudes from intact channels was not observed (Howard et al., 2007). Finally, another study has suggested networks of salt bridges in the D-helix to influence expression of KCNQ currents (Nakajo and Kubo, 2008). These studies frame the idea of a role of the C-terminus in governing KCNQ current amplitudes by controlling channel surface expression.

However, other studies have suggested the C-terminus to not be involved in KCNQ current levels (Etxeberria et al., 2004; Zaika et al., 2008; Choveau et al., 2012b). Replacement of the C-terminus of KCNQ2 subunits with that of KCNQ3 affected surface expression but not current amplitudes when those subunits were co-expressed with KCNQ3 (Etxeberria et al., 2004). Moreover, TIRF microscopy and biotinylation assays showed that divergent current amplitudes of KCNQ channels cannot be explained by differential surface expression (Zaika et al., 2008). As mentioned above, neither the F622L mutation nor the D631S/G633E double mutation, described as critical for channel tetramerization *in vitro*, induced a functional increase in currents from KCNQ3 channels bearing those mutations (Howard et al., 2007). This suggests the D-helix to govern channel oligomerization, but not macroscopic current amplitudes. Recent work from our lab supports this idea (Choveau et al., 2012b). Using native PAGE assays, we found H_2_O_2_ to enhance KCNQ4 channel oligomerization *in vitro* by forming inter-subunit disulfide bonds at position C643 in the D-helix. Indeed, the C643A mutation wholly prevented oligomerization induced by H_2_O_2_ *in vitro*, but only slightly modified KCNQ4 current amplitudes. KCNQ3 channels possess a histidine at the analogous position instead of a cysteine. To probe the role of this cysteine in enabling current amplitudes, we tested the effect of the H646C mutation on KCNQ3 currents. This mutation slightly decreased surface expression, and did not affect KCNQ3 current amplitudes, arguing against a role of the D-helix in controlling KCNQ current amplitudes.

## Networks of interactions in the pore region regulate channel current amplitude

**Figure 1:**
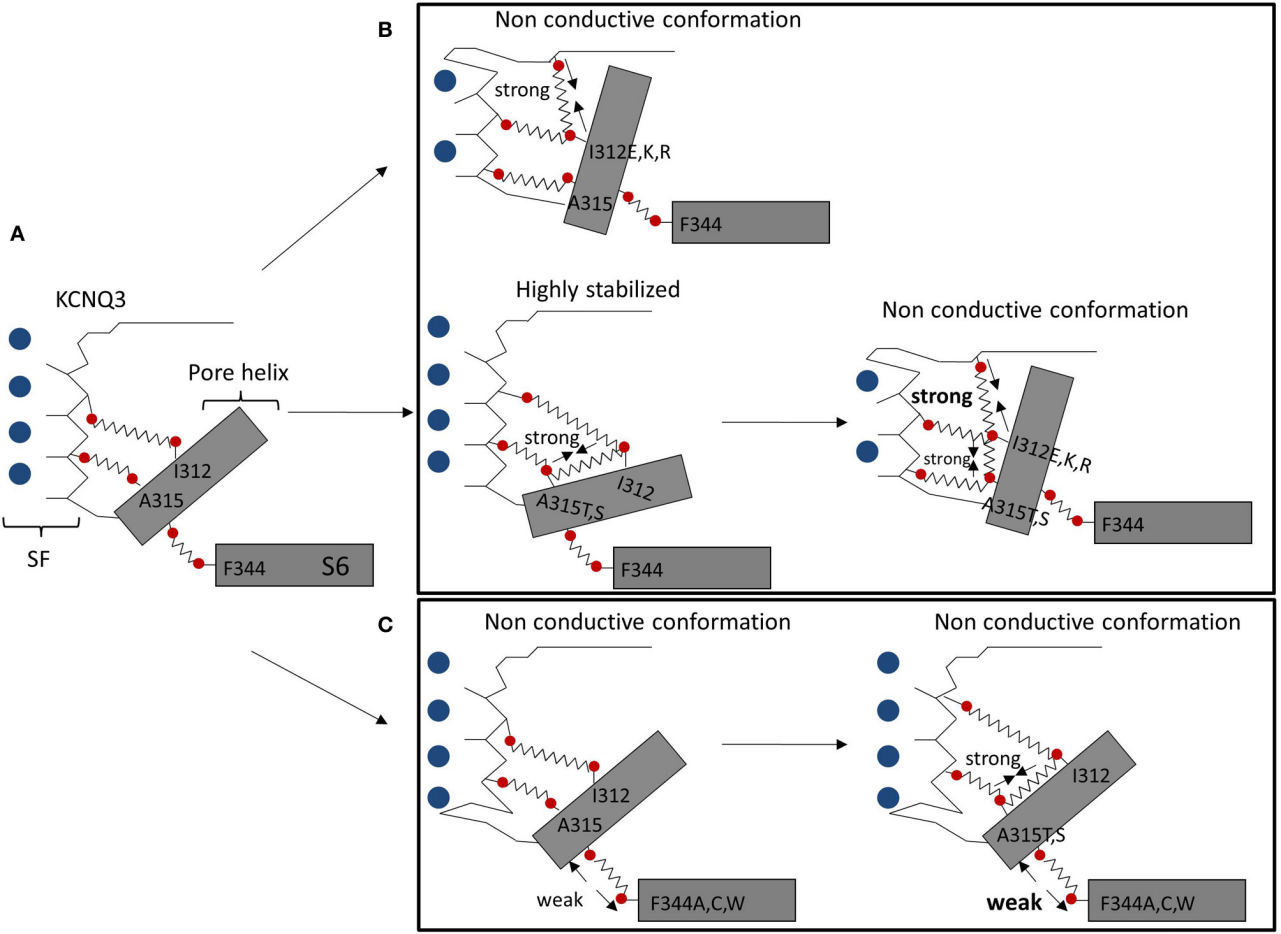
**Networks of interactions in the pore region controlling KCNQ3 gating.** Shown is a schematic representation of the pore region of wild-type KCNQ3 **(A)**. Shown are structural rearrangements resulting from creation of hydrogen bonds between the pore helix and the SF **(B)** or the disruption of the van der Waals interaction between the S6 domain and the pore helix **(C)** (adapted from Choveau et al., 2012b).

Our lab suggests that pore instability is responsible for small KCNQ3 currents, compared to other KCNQ channels. Based on patch-clamp experiments and homology modeling, we identified two networks of interactions between the pore helix and the SF, and between the pore helix and the S6 domain, controlling KCNQ3 gating (Figure 1A). Formation of hydrogen bonds between a hydrophilic residue at position 315 (A315T, S) and the I312 in the pore helix led to ~15-fold increase of current amplitude, compared to wild-type KCNQ3, modeled as stabilizing the SF in a conductive conformation (Zaika et al., 2008) (Figure 1B). In contrast, a hydrophilic residue at position 312 (I312E, I312K, and I312R) was suggested to form destabilizing hydrogen bonds with the top of the SF, affecting the conductive pathway of KCNQ3 and that of KCNQ3 (A315T) (Figure 1B) (Choveau et al., 2012a). Because the residues involved in this network of interactions are highly conserved in KCNQ channels, such interactions could affect the stability of the SF in other KCNQ channels. Indeed, a hydrophilic residue at position 273 (I312 in KCNQ3) in KCNQ2 resulted in a decrease of current amplitude, comparable to that observed in KCNQ3 suggesting similar mechanisms may apply to other KCNQ channels. Interactions between the pore helix and the top of the SF may also promote the channel conductive pathway (Uehara et al., 2008). Indeed, the W309R mutation in the pore helix of KCNQ3 led to a decrease of current compared to wild-type channels that would arise from the destabilization of pore helix-SF interactions. A homology model, based on the crystal structure of Kv1.2, proposed that an arginine (R309), in contrast to a tryptophan residue in wild-type channels, is not close enough to Y319 to make a hydrogen bond. Finally, a second network of interactions between the pore helix and the S6 domain has been postulated as governing KCNQ current amplitudes (Seebohm et al., 2005; Panaghie et al., 2008; Choveau et al., 2012b). In KCNQ3, a phenylalanine (F344) in the S6 domain has been suggested to be close enough to form a van der Waals interaction with the pore helix at A315, stabilizing the conductive pathway (Figure 1C). Disruption of this interaction, by mutating F344 to A, C, or W, resulted in a ~5-fold-decrease of current amplitude compared to wild-type KCNQ3 (Choveau et al., 2012b). As mentioned above, the T315-I312 bond is thought to promote the channel conductive pathway. This stabilizing effect is modeled as abolished by the disruption of the F344-A315 interaction, arguing for a dominant role of this interaction over the T315-I312 bond in KCNQ3 gating (Figure 1C). Interestingly, mutations of the equivalent phenylalanine (F340) in KCNQ1 affect its function as well (Seebohm et al., 2005; Panaghie et al., 2008). The predicted structure of the pore region of KCNQ1, based on the crystal structure of KcsA, suggests this phenylalanine may interact with the pore helix (V310), but with the homologous residue next to A315 in KCNQ3 (Seebohm et al., 2005). This indicates pore helix-S6 interactions might play a role in gating for all KCNQ channels.

## Role of calmodulin in KCNQ channel expression

Many proteins have been described as interacting with the C-terminus. One of those is the ubiquitous calcium sensor, CaM, shown to bind to helices A and B and to be a critical auxiliary subunit of all KCNQ channels tested (Wen and Levitan, 2002; Yus-Najera et al., 2002; Gamper and Shapiro, 2003; Gamper et al., 2005; Etxeberria et al., 2008; Haitin and Attali, 2008; Hernandez et al., 2008; Wiener et al., 2008; Alaimo et al., 2009). CaM seems to be required for channel assembly, but not for channel trafficking (Wen and Levitan, 2002). Indeed, KCNQ2 channels carrying mutations abolishing CaM binding could not produce currents when they were co-expressed with KCNQ3 channels. However, their ability to reach the plasma membrane was not affected, arguing against a role of CaM in channel trafficking. However, CaM may play a role in the exit of channels from the ER (Etxeberria et al., 2008). Indeed, mutant I340E channels, which have a much lower affinity for CaM, are more retained in the ER and overexpression of a KCNQ2 mutant unable to bind CaM increased the fraction of wild-type channels retained in the ER. Finally, the L339R mutation in helix A of KCNQ2, which alters CaM binding to the channel, affected channel transport to the membrane (Alaimo et al., 2009). As described above, CaM binds to helices A and B, so mutations in helix B which disrupt CaM binding to channels should affect channel surface expression. However, cell-surface staining and patch-clamp recordings showed the S511D CaM-binding mutant in helix B to indeed abolish CaM binding to the channel, but not to prevent channels from reaching the plasma membrane (Gomez-Posada et al., 2011). The equivalent mutation in KCNQ3 (A518D) also led to the formation of non-functional channels, suggesting a divergent role of CaM according to KCNQ channel type. Indeed, CaM has been postulated as critical for KCNQ1 assembly (Ghosh et al., 2006; Shamgar et al., 2006), but this seems not to be the case for KCNQ2 (Etxeberria et al., 2008). Moreover, overexpression of CaM reduced current amplitudes of KCNQ2, KCNQ4, and KCNQ5, but not those of KCNQ1 and KCNQ3 (Gamper et al., 2005).

**Figure 2:**
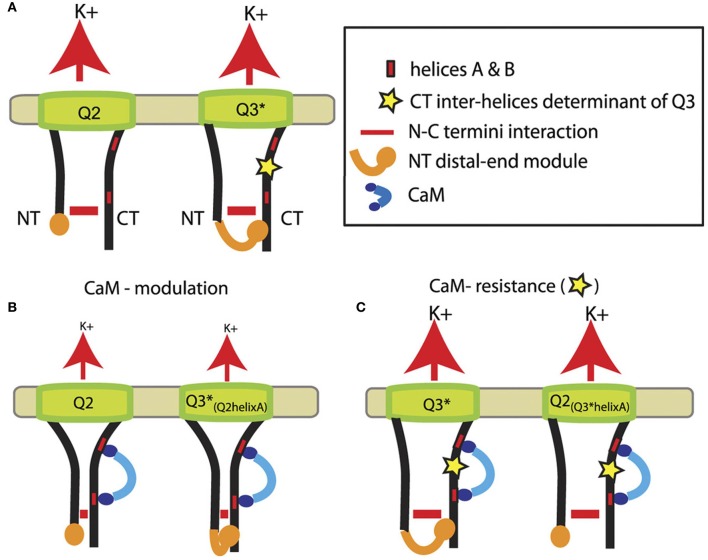
**Model showing the role of N-C terminus interaction in the regulation of KCNQ2-3 channels by CaM. (A)** Representation of KCNQ2 and KCNQ3 mutant (A315T) channels (helices C and D in the C-terminus were omitted for clarity) with the N-C terminus interaction (horizontal red lines) and the additional interaction between the distal end of the N-terminus and the C-terminus (orange) in KCNQ3 mutant (A315T) and probably wild-type KCNQ3 channels. **(B)** CaM binding induces downregulation of current amplitudes, which is depicted by smaller K^+^ fluxes (red arrows), accompagnied by a stronger N-C terminus interaction. **(C)** Helix A of KCNQ3 confering resistance to CaM on channel function (adapted from Etzioni et al., 2011).

Another study suggests CaM to be involved in intramolecular regulation involving N-C termini interactions (Etzioni et al., 2011). Based on pull-down assays, FRET analysis and patch-clamp experiments, a model has been proposed depicting the regulation of KCNQ2 and KCNQ3 channels by CaM (Figure 2). In wild-type KCNQ2 and KCNQ3 (A315T) channels, an N-C termini interaction occurs (Figure 2A). An additional interaction is formed in KCNQ3 (A315T) between the distal part of the N-terminus and the C-terminus (Figure 2A, left). In that model, CaM binding to helices A and B downregulates wild-type KCNQ2, which is accompanied by a stronger N-C termini interaction (Figure 2B). Similar results were found in a chimeric KCNQ3 channel containing helix A of KCNQ2, showing that the interaction between the distal ends of the N-C termini does not underlie downregulation of channels by CaM (Figure 2B, left). In contrast, replacement of helix A of KCNQ2 by that of KCNQ3 rendered channels resistant to CaM, preventing closer N-C terminus proximity as in KCNQ3 (A315T) channels (Figure 2C).

In summary, three regions have been postulated as controlling KCNQ channel expression. The N-terminus plays a role in the open probability and KCNQ channel trafficking to the membrane. The C-terminus appears to be critical for KCNQ surface expression, assembly and tetramerization, but seems to play only a secondary role compared to the pore region, in governing KCNQ current amplitudes. In this region of KCNQ3, two networks of interactions, affecting the stability of SF, between the pore helix and the SF, and between the S6 domain and the pore helix, have been highligthed. KCNQ channels play important roles in neuronal excitability and also in the heart, smooth muscle and epithelia. Dysfonction of these channels leads to many diseases such as epilepsy and cardiac arrthymias. The fuller understanding of the mechanims regulating the activity of KCNQ channels may allow the development of new therapies against these diseases by targeting these three regions. However, many questions have not been solved. Could such networks of pore interactions affect the stability of SF in other voltage-gated channels? Are the effects of the N-C termini on channel surface expression additive or cooperative? By what molecular mechanisms do the N-C termini regulate channel trafficking? How can we integrate results showing dual roles of CaM suggested for KCNQ channels in Ca^2+^ sensing and membrane expression? We look forward to continued work on these issues.

### Conflict of interest statement

The authors declare that the research was conducted in the absence of any commercial or financial relationships that could be construed as a potential conflict of interest.
